# Efficacy of a Salmonella Bacteriophage Cocktail Against Multidrug‐Resistant Salmonella Isolates From Clinical and Food Samples

**DOI:** 10.1002/hsr2.72439

**Published:** 2026-04-29

**Authors:** Taras Gabisonia, Manana Loladze, Maia Zarnadze, Natia Tamarashvili, Natela Chakhunashvili, Manana Nadiradze, Maia Alibegashvili, Tatiana Eliava, Tamar Kalandarishvili, Teimuraz Katamadze

**Affiliations:** ^1^ Petre Shotadze Tbilisi Medical Academy Tbilisi Georgia; ^2^ G. Eliava Institute of Bacteriophages, Microbiology and Virology Tbilisi Georgia; ^3^ New Vision University Tbilisi Georgia

## Abstract

**Introduction:**

*Salmonella* is a significant foodborne pathogen responsible for severe gastroenteritis and systemic infections in humans and animals. The increasing prevalence of antimicrobial‐resistant *Salmonella* strains poses a major public health challenge. This study aimed to investigate the antimicrobial resistance patterns of *Salmonella* isolates obtained from various sources, including food and human clinical samples, and to study the effectiveness of bacteriophages against isolated strains.

**Materials and Methods:**

A total of 80 *S. Typhimurium* isolates were obtained from two clinics between 2015 and 2022, and 16 ground meat samples were collected from agricultural markets. Standard microbiological methods were used for *Salmonella* isolation, including culture‐based techniques and biochemical confirmation. The antimicrobial susceptibility of the isolates was determined using the disk diffusion method using 8 common antibiotics. New bacteriophages were isolated against multidrug‐resistant *Salmonella* isolates. Electron microscopy for bacteriophage morphology investigation was employed.

**Results:**

Antimicrobial susceptibility testing revealed high resistance rates to tetracycline, streptomycin, ampicillin, chloramphenicol and ciprofloxacin, with multidrug resistance (MDR) observed in 16% of the isolates. The phage cocktail composed of six phages showed efficacy against 88.3% *S. Typhimurium* strains of different origins.

**Conclusion:**

The study highlights the widespread occurrence of antimicrobial‐resistant *Salmonella* in food and human samples. The presence of MDR strains poses a serious threat to public health, necessitating biosecurity interventions to limit the spread of resistant *Salmonella* strains. The bacteriophages assessed in this study demonstrated significant potential for the biocontrol of *Salmonella* strains. The phage cocktail may be used for both food safety and therapeutic purposes, particularly against antibiotic‐resistant bacteria causing salmonellosis.

## 1 Introduction

*Salmonella* is a significant zoonotic foodborne pathogen, responsible for 93.8 million cases of gastroenteritis and 155,000 deaths annually [1]. It is one of the major causes of enteric infections worldwide, often transmitted through contaminated animal products such as eggs, meat, and poultry. *Salmonella Typhimurium* is a major cause of foodborne illness and poses economic challenges to both humans and animals [1, 2, 3].

Antimicrobial resistance is an escalating global health concern, leading to an increased frequency of bacteremia, urinary tract infections, pneumonia, and endocarditis as a result of *S. Typhimurium* infection. Multidrug resistance to common antibiotics complicates the treatment options of *Salmonella* infections. In the U.S., *Salmonella* remains a leading cause of foodborne infections, with an increasing number of cases showing resistance to antibiotics like ampicillin, streptomycin, and tetracycline [4].

In Europe, there has been a rise in MDR *Salmonella*, especially strains resistant to fluoroquinolones and third‐generation cephalosporins. A significant outbreak of MDR *S. Typhimurium* across 10 countries affected 150 people, predominantly young children, with the strain showing resistance to six antibiotic classes and in veterinary practices [5].

The increasing prevalence of MDR *Salmonella* highlights the urgent need for both new antibiotics and alternative therapies, such as bacteriophages. Bacteriophages are one such tool, widely used in various fields, including medicine and agriculture. The main advantages of phage therapy over traditional antibiotic treatment are as follows: Phages are highly effective against multidrug‐resistant pathogenic bacteria and are generally safer, as they do not disrupt the normal microbial flora—phages specifically target and eliminate only the harmful bacteria. Moreover, phages can quickly respond to the emergence of resistant bacterial strains due to their relatively high mutation rates compared to those of bacteria. Side effects associated with phage therapy are extremely rare, and the vast diversity of naturally occurring phages offers an almost unlimited pool for therapeutic applications. Phage application is reported to be highly efficient, highly specific, and cost‐effective when applied for pathogen eradication in food products and water, and in veterinary. Bacteriophages have demonstrated high effectiveness against foodborne pathogenic microorganisms [6, 7, 8, 9, 10, 11, 12, 13]. *Salmonella* phages may be used in the following ways to biocontrol foodborne pathogens: pre‐harvest pathogen control in livestock and poultry reared for food production; decontamination of inanimate surfaces in food processing facilities; and post‐harvest foodborne pathogen control by directly applying phages to harvested or processed foods [14, 15, 16, 17]. As a result, reducing *Salmonella* in the food chain will contribute to a consistent decline in related human infections.

The present study aims to investigate the antibiotic resistance profiles of *Salmonella* strains isolated from both human samples and food products, and to develop a phage cocktail effective against these strains, including multidrug‐resistant (MDR) isolates from both sources.

## 2 Materials and Methods

### 2.1 Bacterial Strains

A total of 80 clinical isolates of *Salmonella Typhimurium* were obtained between 2015 and 2022 from two hospitals in Tbilisi, Georgia. These bacterial strains were isolated from patient stool samples and studied in the hospital's laboratories. All strains were identified as *Salmonella Typhimurium* by the respective hospital laboratories. To protect patient identities, microbiologists at these hospitals assigned codes to the strains. All strains were confirmed as *Salmonella Typhimurium* by us using culture‐based microbiological, biochemical, and serological methods, which we employed for the identification of *Salmonella* strains from food samples.

Additionally, 14 *Salmonella Typhimurium* strains were isolated from 16 ground meats purchased from agricultural markets near Tbilisi.

### 2.2 Isolation of *Salmonella Typhimurium* From Food Samples (Ground Meat)

**Sample Collection and Preparation:** A total of 16 ground meat samples were collected from various agricultural markets. The samples were homogenized, and 25 g of each sample was transferred into 225 mL of buffered peptone water (BPW) (Oxoid, UK) and mixed thoroughly.

**Enrichment:** The meat sample suspension was incubated at 37°C for 24 h to allow potential *Salmonella* cells to proliferate.

**Subculture onto Selective Agar:** After incubation, 100 µL of the enriched suspension was streaked onto SS (*Salmonella*, *Shigella*) agar and XLD agar and incubated at 37°C for 24–48 h.

**Identification of** ***Salmonella*** **Colonies:** Colonies presumptive for *Salmonella* were identified by colony morphology on SS and XLD agars. Colonies that appeared typical of *Salmonella* were selected for further analysis.

**Biochemical and Serological Confirmation:** Presumptive *Salmonella* colonies were biochemically identified through API 20E. *Salmonella* species were confirmed by serological methods using specific antisera for *Salmonella Typhimurium* (Bio‐Rad) (in accordance with established protocols. *Salmonella* serogroups were identified serologically through a slide agglutination test, employing both standard polyvalent and monovalent *Salmonella* antisera (Bio‐Rad). This method allowed for the precise classification of the strains based on their antigenic properties [18].

### 2.3 Antibiotics Susceptibility Test for *S. Typhimurium* Strains

Three to five bacterial colonies were transferred into 10 ml Brain Heart Infusion Broth (Oxoid), incubated at 37°C for 24 h, diluted 1:10 in 9 ml of 0.1% Peptone water (10 g of peptone and 5 g of Sodium chloride/L), and spread over the surface of a dried Mueller‐Hinton Agar (MHA) plate using a cotton swab. The MHA plates were kept at room temperature for 10 min to allow absorption of excess liquid. Antibiotic discs were placed over the surface of each inoculated plate (four discs per plate). After incubation for 24 h at 37°C, the diameter of the growth inhibition zone around each disc was measured, and in accordance with the resultant diameter, each strain was identified as susceptible (S), intermediate (I), or resistant (R) in accordance with the European Committee on Antimicrobial Susceptibility Testing (EUCAST) [19]. The following antibiotics were used in *S. Typhimurium* tests: Ampicillin −10 µg; Chloramphenicol − 30 µg; Gentamicin −10 µg; Streptomycin − 10 µg; Ciprofloxacin − 5 µg; Tetracycline − 30 µg; Trimethoprim − 5 µg; Kanamycin − 30 µg.

### 2.4 Isolation of *Salmonella* Bacteriophages From Sewage

From the investigated strains, 3 human and 3 food multidrug‐resistant strains were used as host strains for the isolation and investigation of bacteriophages.

Sewage samples were filtered through a 0.45‐µm Millipore filters, and the filtrates were examined for the presence of phages. 90 ml of each filtrate was enriched with 10 ml of concentrated broth, and added to 1 ml of 18‐h culture of the target bactria. Then, the whole mixture was incubated at 37°C. After 18–24 h, the incubated material was centrifuged at 9000 g for 15 min to remove debris and then filtered through 0.22 µm Millipore filters. The presence of phage was determined by spotting methods.

### 2.5 Spotting Assay

The host bacteria were grown overnight, and then 100 μl of cell suspension was added to 5 ml of soft LB agar (0.6% agar), which had been pre‐heated to 42°C in a water bath. The resultant mixture was gently vortexed, poured over LB agar plates (1.5% agar), and allowed to solidify at room temperature during 30 min to produce bacterial lawns. Then, 10 μl of phage stock dilutions (10‐fold serial dilutions in SM buffer) were spotted onto the upper agar layer, and the plates were dried at room temperature for 30 min. Then, the plates were incubated overnight at 37°C, and inspected for single plaques or bacterial growth inhibition zones after 24 h [20].

### 2.6 Preparation of Concentrated Phage Stocks (Bilayer Agar Method)

A total of 100 μl of the host bacterium culture grown overnight and 1 ml of phage (10^3^PFU/ml) were mixed; then 3 ml of molten soft‐agar (0.7%) was added to each tube, maintained at 45°C, and the mixture was gently vortexed and poured over LB agar plates (1.5% agar). The Petri dishes were incubated at 37°C. After 18–20 h incubation, 3 ml of broth was spread over the agar and left for 15–20 min. Using a spreading rod or spatula, the soft agar with broth was scraped and transferred to a centrifuge tube, and centrifuged at 6000 g for 45 min. The supernatant was filtered through a 0.22 nm filter, transferred into the sterile vial and titrated. Plaques were counted, and titers were expressed as plaque‐forming units per milliliter (PFU/mL). All assays were performed in triplicate. Plaque morphology (size, clarity, halo formation) was recorded after incubation.

#### 2.6.1 Phage Purification

Individual well‐isolated plaques were selected using sterile pipette tips and suspended in 500 µL of SM buffer. The suspension was vortexed and incubated at 4°C for 2–4 h to allow phage diffusion. The eluate was centrifuged briefly and filtered (0.22 µm).

To ensure purity, at least three successive rounds of plaque purification were performed using the double‐layer agar technique. High‐titer lysates were obtained by propagating purified phages on their respective host strains and harvesting lysates after complete lysis.

### 2.7 Study of the Host Range of Bacteriophage and Selection of the Most Efficient Phage

The phages were investigated for host range specificity and lysis efficiency against *Salmonella* strains. Each strain was inoculated on Tryptic Soy Agar (TSA), and 10 μl of phages (1 × 10^7^ PFU ml^−1^) was dropped over the plate with the inoculated culture. The plates were then incubated during 18 h at 37°C, and the presence of plaques was observed.

### 2.8 Characterization of Selected *Salmonella* Bacteriophages By Electron Microscopy

Pure phage stocks with titers of ~10^9^ PFU/ml were prepared. A sample of each stock was stained negatively with 1% uranyl acetate, and electron microphotographs were taken at various magnifications. Based on the morphology of the phage, all phages were classified into their respective family according to the International Committee on Taxonomy of Viruses [3].

### 2.9 Statistical Analysis

All experiments were conducted in triplicate. To evaluate whether the observed difference in lytic efficacy was statistically significant, a **two‐sample *z*‐test for proportions** was performed. A *p*‐value of less than 0.05 (*p* < 0.05) was considered statistically significant.

## 3 Results

### 3.1 Antimicrobial Susceptibility Testing of Clinical and Food Strains of ***Salmonella** Typhimurium*

A total of 80 clinical strains and 14 food‐derived strains of *Salmonella Typhimurium* were tested for susceptibility to eight commonly used antibiotics in clinical and veterinary practice. The results revealed that the majority of *Salmonella* strains exhibited resistance to the antibiotics tested (Table 1).

**Table 1:** Antimicrobial Susceptibility Pattern of *Salmonella Typhimurium*.

Antibiotic group	Antibiotic	Antibiotic susceptibility					
		Susceptible		Intermediate		Resistant	
		*N*	%	*N*	%	*N*	%
Aminoglycosides	Streptomycin	56	59.6	16	17	22	23.4
	Gentamicin	64	68.1	10	10.6	20	21.3
	Kanamycin	57	60.1	20	21.3	17	18.1
Phenicol	Phenicol	53	56.4	25	26.6	16	17
Tetracyclines	Tetracycline	53	56.4	13	13.8	28	29.8
β‐Lactams	Ampicillin	60	63.8	17	18.1	17	18.1
Diaminopyrimidine	Trimethoprim	67	71.3	16	17	11	11.7
Quinolones	Ciprofloxacin	47	50	32	34	15	15.9

Resistance in clinical strains was most frequent to tetracycline (26.25%), streptomycin (22.5%), gentamycin (21.25%), ampicillin (18.75%), kanamycin (17.5%), phenicol and ciprofloxacin (15%), followed by lower levels of resistance to trimethoprim (11.25%). Food isolates were resistant most frequently to tetracycline (46.6%), phenicol and streptomycin (26.6%), ciprofloxacin, gentamycin and kanamycin (20%), followed by lower levels of resistance to ampicillin and trimethoprim (13.3%). From investigates 94 strains 9 clinical and 6 food strains were characterized with resistance to 3 or more different group of antibiotics (Table 2).

**Table 2:** Antibiotic Resistance Pattern of the MDR *Salmonella* Strains.

*N*	Strains	Source	Antibiotic Resistance Profiles		
			3	4	5
1	*S. Typhimurium #1*	Clinical isolate			Amp, Chl, Str, Cip, Tmp
2	*S. Typhimurium #5*	Clinical isolate		Amp, Chl, Tet, Cip	
3	*S. Typhimurium #8*	Clinical isolate	Chl, Str, Tet		
4	*S. Typhimurium #12*	Clinical isolate	Chl, Tet, Gen		
5	*S. Typhimurium #13*	Clinical isolate			Amp, Str, Tet, Cip, Tmp
6	*S. Typhimurium #15*	Clinical isolate	Str, Tet, Cip		
7	*S. Typhimurium* #24	Clinical isolate	Chl, Str, Gen		
8	*S. Typhimurium* #38	Clinical isolate	Tet, Cip, Gen		
9	*S. Typhimurium* #40	Clinical isolate		Amp, Chl, Tet, Cip	
10	*S. Typhimurium* #21	Ground meat		Amp, Chl, Str, Tet	
11	*S. Typhimurium* #22	Ground meat	Chl, Str, Tmp		
12	*S. Typhimurium* #25	Ground meat		Str, Tet, Cip, Tmp	
13	*S. Typhimurium* #26	Ground meat	Amp, Chl, Tet		
14	*S. Typhimurium* #27	Ground meat	Str, Tet, Cip		
15	*S. Typhimurium* #31	Ground meat		Tet, Cip, Kan, Tmp	

Abbreviations: Amp, Ampicillin; Chl, Chloramphenicol; Cip, Ciprofloxacin; Gen, Gentamicin; Kan, Kanamycin; Str, Streptomycin; Tet, Tetracycline; Tmp: Trimethoprim.

### 3.2 Isolation of Bacteriophages

To isolate bacteriophages effective against newly isolated pathogenic *Salmonella Typhimurium* strains, we analyzed 40 samples collected from sewage, river water, and other environmental sources. Sixteen of these samples contained phages capable of lysing pathogenic *S. Typhimurium* strains. From these, six phages with the ability to infect and lyse a broad range of *Salmonella* strains—particularly multidrug‐resistant (MDR) isolates—were selected for further study: vB_Stm 16, vB_Stm 17, vB_Stm 18, Sal phi 21, vB_Stm 29, and Sal phi 13 (Table 3).

**Table 3:** Host Strains and Sources of Selected Bacteriophages.

#	Bacteriophage	Source of bacteriophages	Host strains	Source of host strains
1	vB_Stm 16	Sewage water “Rioni”	*S. Typhimurium #8*	Stool samples
2	vB_Stm 17	Sewage water “Mtkvari”	*S. Typhimurium #15*	Stool samples
3	vB_Stm 18	Sewage water “Rioni”	*S. Typhimurium #24*	Stool samples
4	vB_Stm 21	Sewage water “Mtkvari”	*S. Typhimurium #26*	Ground meat
5	vB_Stm 29	Sewage water “Mtkvari”	*S. Typhimurium #22*	Ground meat
6	Sal. phi 13	Sewage water “Mtkvari”	*S. Typhimurium* #31	Ground meat

**Figure 1:**
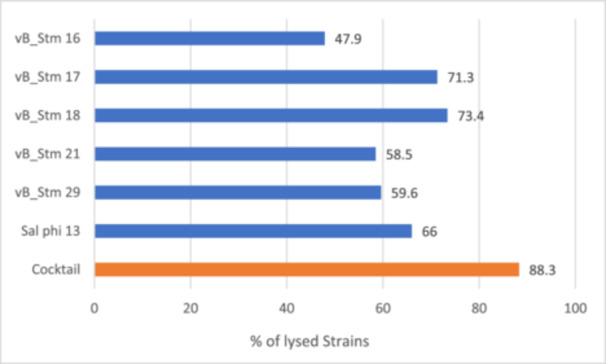
The efficacy of individual bacteriophages and phage cocktails against *S. Typhimurium* strains isolated from clinical and food samples.

The lytic activity of six individual bacteriophages and a phage cocktail was evaluated against *Salmonella enterica* serovar *Typhimurium* strains isolated from both clinical (human) and food sources (Table 4, Figure 1). Individual phages demonstrated variable lysis profiles, with efficacy ranging from 47.9% to 73.4%, depending on the phage and the strain tested. Among these, phage vB_Stm 18 showed the highest individual activity, lysing 73.4% of the tested strains. In contrast, the phage cocktail—composed of six partially complementary individual phages—demonstrated broader lytic activity, lysing 88.3% of *Salmonella Typhimurium* strains overall. To assess whether this difference in efficacy was statistically significant, a two‐sample *z*‐test for proportions was performed. The result (*z* = 0.33, *p* = 0.744) indicated no statistically significant difference between the two groups, suggesting that the phage cocktail shows strong lytic activity against *Salmonella* strains from different sources.

**Table 4:** A Host Range of Selected *Salmonella* Phages and Phage Cocktail.

	Strains	Antibiotics resistance profiles	Lysis by phage*						
			*vB_Stm 16*	*vB_Stm 17*	*vB_Stm 18*	*vB_Stm 21*	*vB_Stm 29*	*Sal phi 13*	*Cocktail*
1	*S. Typhimurium #1*	Amp, Chl, Str, Cip, Trm							
2	*S. Typhimurium #5*	Amp, Chl, Tet, Cip							
3	*S. Typhimurium #8*	Chl, Str, Tet							
5	*S. Typhimurium #13*	Amp, Str, Tet, Cip, Tmp							
6	*S. Typhimurium #15*	Str, Tet, Cip, Gen, Kan							
7	*S. Typhimurium #20*	Str, Tet							
8	*S. Typhimurium* #23	Chl, Str							
9	*S. Typhimurium* #24	Chl, Str, Gen							
10	*S. Typhimurium* #28	Str							
11	*S. Typhimurium* #29	Amp, Chl							
12	*S. Typhimurium* #30	Tet, Cip							
13	*S. Typhimurium* #32	Gen, Tmp							
14	*S. Typhimurium* #33	Tmp							
15	*S. Typhimurium* #38	Tet, Cip, Gen							
16	*S. Typhimurium* #40	Amp, Chl, Tet, Cip							
17	*S. Typhimurium* #46	Chl, Gen							
18	*S. Typhimurium* #48	Amp, Chl							
19	*S. Typhimurium* #50	Tet, Kan							
20	*S. Typhimurium* #51	Gen, Kan							
21	*S. Typhimurium* #52	Gen, Tmp							
22	*S. Typhimurium* #53	Str, Tmp							
23	*S. Typhimurium* #54	Str, Tet							
24	*S. Typhimurium* #55	Str, Tet							
25	*S. Typhimurium* #56	Tet, Cip							
26	*S. Typhimurium* #57	Tet							
27	*S. Typhimurium* #58	Tet, Cip							
28	*S. Typhimurium* #59	Amp, Chl							
29	*S. Typhimurium* #99	Cip, Gen							
30	*S. Typhimurium* #100	Tet, Cip							
31	*S. Typhimurium* #101	Str, Gen							
32	*S. Typhimurium* #102	Tmp, Kan							
33	*S. Typhimurium* #103	Str, Gen							
34	*S. Typhimurium* #104	Kan							
35	*S. Typhimurium* #105	Amp							
36	*S. Typhimurium* #106	Tet, Kan							
37	*S. Typhimurium* #107	Amp, Tet							
38	*S. Typhimurium* #108	Str							
39	*S. Typhimurium* #109	—							
40	*S. Typhimurium* #110	—							
41	*S. Typhimurium* #111	—							
42	*S. Typhimurium* #112	Kan							
43	*S. Typhimurium* #113	Amp							
44	*S. Typhimurium* #114	—							
45	*S. Typhimurium* #115	Amp, Gen							
46	*S. Typhimurium* #116	Str							
47	*S. Typhimurium* #117	—							
48	*S. Typhimurium* #118	Str							
49	*S. Typhimurium* #119	Kan							
50	*S. Typhimurium* #120	Amp							
51	*S. Typhimurium* #121	Kan							
52	*S. Typhimurium* #122	Amp, Kan							
53	*S. Typhimurium* #123	Str, Gen							
54	*S. Typhimurium* #124	—							
55	*S. Typhimurium* #125	Gen, Tmp							
56	*S. Typhimurium* #126	—							
57	*S. Typhimurium* #127	—							
58	*S. Typhimurium* #128	—							
59	*S. Typhimurium* #129	—							
60	*S. Typhimurium* #130	—							
61	*S. Typhimurium* #131	Amp							
62	*S. Typhimurium* #132	Tet							
63	*S. Typhimurium* #133	Gen							
64	*S. Typhimurium* #134	—							
65	*S. Typhimurium* #135	—							
66	*S. Typhimurium* #136	Str							
67	*S. Typhimurium* #137	—							
68	*S. Typhimurium* #138	Amp							
69	*S. Typhimurium* #139	—							
70	*S. Typhimurium* #140	Tmp, Kan							
71	*S. Typhimurium* #141	—							
72	*S. Typhimurium* #142	Cip, Gen							
73	*S. Typhimurium* #143	Kan							
74	*S. Typhimurium* #144	Gen							
75	*S. Typhimurium* #145	Tet							
76	*S. Typhimurium* #146	—							
77	*S. Typhimurium* #147	Chl							
78	*S. Typhimurium* #148	Tet							
79	*S. Typhimurium* #149	—							
80	*S. Typhimurium* #150	—							
81	*S. Typhimurium* #21	Amp, Chl, Str, Tet, Cip							
82	*S. Typhimurium* #22	Chl, Str, Gen, Tmp							
83	*S. Typhimurium* #25	Str, Tet, Cip, Tmp, Kan							
84	*S. Typhimurium* #26	Amp, Chl, Tet							
85	*S. Typhimurium* #27	Str, Tet, Cip							
86	*S. Typhimurium* #31	Tet, Cip, Gen, Tmp, Kan							
87	*S. Typhimurium* #77	Tet							
88	*S. Typhimurium* #79	—							
89	*S. Typhimurium* #82	Chl							
90	*S. Typhimurium* #85	Tet							
91	*S. Typhimurium* #87	—							
92	*S. Typhimurium* #91	—							
93	*S. Typhimurium* #93	—							
94	*S. Typhimurium* #95	—							

* Dark zones correspond to complete lysis of the bacterial lawn; transparent zones correspond to no lysis.

**Figure 2:**
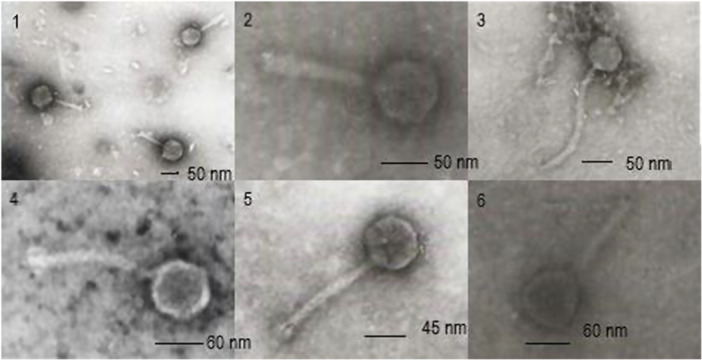
TEM images of bacteriophages: 1‐vB_Stm 16, 2‐vB_Stm 17, 3‐vB_Stm 18, 4‐vB_Stm 21, 5‐Sal.phi13, 6‐vB_Stm 29.

Morphological characteristics of the six phages were examined, and the TEM images showed that two phages vB_Stm 18, and Sal phi 13, were members of the *Siphoviridae* family. Siphoviruses have an icosahedral head that measures approximately 45–60 nm in diameter. Both phages contained a long, non‐contractile tail with a length of 190 nm for vB_Stm 18 and 100 nm for Sal phi 13. On the other hand, four phages vB_Stm 16, vB_Stm 17, vB_Stm 21, and vB_Stm 29, were classified as myoviruses, where they belong to the *Myoviridae* family, per the criteria established by the International Committee on Taxonomy of Viruses. The TEM image of vB_Stm 16, vB_Stm 17 phages showed that they contained a round head in shape with isometric symmetry (50–60 nm in diameter) and a contractile sheathed tail (95 nm in length for vB_Stm 17 and 75 nm for vB_Stm 16), and vB_Stm 21 and vB_Stm 29 phages had icosahedral head with 54–60 nm in diameter and a contractile sheathed tail 120 nm in length for vB_Stm 21 and 95 nm in length for vB_Stm 29 (Table 5, Figure 2).

**Table 5:** Morphological Characteristics of Selected *Salmonella* Phages.

Bacteriophage	Head diameter, nm	Head length, nm	Tail diameter, nm	Tail length, nm	Family
vB_Stm 16	60	60	10	75	*Myoviridae*
vB_Stm 17	50	60	20	95	*Myoviridae*
vB_Stm 18	50	50	15	190	*Siphoviridae*
vB_Stm 21	54	54	18	120	*Myoviridae*
Sal.phi13	45	45	15	100	*Siphoviridae*
vB_Stm 29	60	50	15	95	*Myoviridae*

## 4 Discussion

This study demonstrates the high prevalence of antimicrobial resistance among *Salmonella Typhimurium* isolates from both clinical and food sources in Georgia. Resistance was most pronounced against tetracycline, streptomycin, ampicillin, chloramphenicol, and ciprofloxacin, consistent with global reports of widespread resistance to these antibiotics in *Salmonella* due to their extensive use in human and veterinary medicine. The detection of multidrug‐resistant (MDR) strains in 16% of isolates highlights the growing public health threat, particularly given the presence of MDR strains in food samples, which suggests a direct transmission route from contaminated food products to humans.

Our findings align with previous reports from Europe and North America, where MDR *Salmonella* strains resistant to fluoroquinolones and β‐lactams have been increasingly documented. The resistance patterns observed here—especially against tetracycline and streptomycin—likely reflect the continued reliance on these antibiotics in agriculture, which contributes to persistence of resistant strains in the food chain. This underscores the urgent need for stricter antimicrobial stewardship and biosecurity measures [21].

The bacteriophage cocktail developed in this study showed strong lytic activity, effectively targeting 88.3% of *S. Typhimurium* strains, including MDR isolates. This broad host range is significant, as phage therapy has been proposed as a viable alternative or complement to antibiotics in both therapeutic and food safety applications. The inclusion of phages isolated from diverse environmental sources likely contributed to the cocktail's efficacy, reflecting the adaptability and diversity of naturally occurring phages. Similar studies have demonstrated that phage cocktails can control MDR *Salmonella* infections in poultry and reduce bacterial loads in food matrices [22, 23].

Phage therapy offers several advantages over conventional antibiotics. Unlike broad‐spectrum antimicrobials, bacteriophages specifically target pathogenic bacteria without disturbing commensal microbiota, thereby reducing collateral damage. Their ability to co‐evolve with bacterial hosts provides a dynamic mechanism to counteract resistance development. Importantly, phage‐based interventions have been shown to be safe in both animal and human applications, with rare adverse effects. The demonstrated effectiveness of phages against foodborne *Salmonella* suggests potential applications in pre‐harvest pathogen control, food processing environments, and direct food decontamination [24, 25].

In conclusion, this study highlights the dual challenge posed by MDR *Salmonella* strains in clinical and food settings, while also demonstrating the potential of bacteriophage cocktails as effective biocontrol agents. Integrating phage therapy into food safety protocols and clinical practice could significantly reduce the burden of antibiotic‐resistant *Salmonella* infections [26]. Future research should focus on optimising phage formulations, evaluating their stability under real‐world conditions, and exploring synergistic applications with existing antimicrobial strategies.

## 5 Conclusion

This study reveals the significant antimicrobial resistance of *Salmonella Typhimurium* in both clinical and food sources. All *S. Typhimurium* strains showed multidrug resistance to three or more antibiotics of different groups: ciprofloxacin, tetracycline, gentamycin, trimethoprim and chloramphenicol. These resistance patterns highlight serious challenges in current treatment strategies for *Salmonella* infections and emphasize concerns about food safety and the risk of transmission to humans. In response to this growing threat, a bacteriophage cocktail formulated using six of the most effective phages was developed. This cocktail showed high efficacy, successfully targeting 88.75% of clinical isolates and 87.5% of food‐derived strains, indicating strong potential as an alternative therapeutic approach against multidrug‐resistant *Salmonella* infections. Given the high lytic activity and broad spectrum of this phage cocktail, it represents a promising alternative for the prevention and treatment of salmonellosis caused by multidrug‐resistant *Salmonella* pathogens. This approach could enhance therapeutic options and address the challenges posed by antibiotic resistance in Salmonella infections.

The therapeutic use of phage cocktails—combinations of two or more phage types to create pharmacologically diverse formulations—is gaining increasing attention. The main advantage of using phage cocktails lies in their broader spectra of activity in comparison to individual phage isolates: they can target a wider range of bacterial types and remain effective under more varied conditions. Overall, this approach offers greater potential for presumptive or empirical treatment compared to single phage applications. Additionally, phage cocktails are believed to have a lower likelihood of inducing microbial resistance [7, 8].

This research was conducted at P. Shotadze Tbilisi Medical Academy, the G. Eliava Institute of Bacteriophage, Microbiology and Virology, Laboratory of Applied Microbiology and New Vision University.

## Author Contributions

**Manana Loladze:** conceptualization, investigation, writing – original draft, methodology, validation, resources. **Maia Zarnadze:** methodology, validation, visualization, data curation, investigation. **Natia Tamarashvili:** methodology, visualization, data curation, investigation, supervision. **Natela Chakhunashvili:** methodology, visualization, data curation, investigation. **Manana Nadiradze:** investigation, methodology, visualization. **Maia Alibegashvili:** investigation, methodology, visualization. **Tatiana Eliava:** investigation, methodology, visualization. **Tamar Kalandarishvili:** investigation, methodology, visualization. **Teimuraz Katamadze:** investigation, methodology, visualization. **Taras Gabisonia:** conceptualization, research, overall management and supervision, writing the manuscript, reviewing the manuscript.

## Ethics Statement

The activities and their related tasks described in this paper were approved (IRB approval #04‐11052023) by the Bioethics International Committee of the Petre Shotadze Tbilisi Medical Academy.

## Consent

All affiliated institutions and authors express consent for publication.

## Conflicts of Interest

The authors declare no conflicts of interest.

## Transparency Statement

The lead author, Gabisonia T, affirms that this manuscript is an honest, accurate, and transparent account of the study being reported; that no important aspects of the study have been omitted; and that any discrepancies from the study as planned (and, if relevant, registered) have been explained.

## Data Availability

The data that support the findings of this study are available in the Supporting Information of this article.
